# Response to ‘Serum level of adiponectin is a surrogate independent biomarker of radiographic disease progression in early rheumatoid arthritis: results from the ESPOIR cohort’ – authors’ reply

**DOI:** 10.1186/ar4538

**Published:** 2014-04-10

**Authors:** Jérémie Sellam, Soraya Fellahi, Jean-Philippe Bastard, Jacqueline Capeau, Francis Berenbaum

**Affiliations:** 1 Service de Rhumatologie, Hôpital Saint-Antoine, 184 rue du Faubourg Saint-Antoine, 75012 Paris, France; 2 Inflammation–Immunopathology–Biotherapy i2B Department, 184 rue du Faubourg Saint-Antoine, 75012 Paris, France; 3 UPMC Université Paris 06, 184 rue du Faubourg Saint-Antoine, 75012 Paris, France; 4 INSERM, UMR_S 938, Faculté de Médecine 184 rue du Faubourg Saint-Antoine, 75012 Paris, France; 5 AP-HP, Hopital Tenon, Department of Biochemistry, 1 rue de la Chine, 7520 Paris, France

## 

We thank Dr Toussirot for his interest [1] in our work demonstrating that serum-level adiponectin is associated with subsequent radiographic progression in early rheumatoid arthritis (RA) [2]. We would like to respond to each comment.

First, our objective was not to determine whether the serum adipokine level might reflect RA disease activity cross-sectionally, but was to find surrogate markers able to predict structural radiographic progression. We performed such an analysis and found no correlation between any serum adipokine levels and the disease activity score in 28 joints (data not shown).

Concerning the second and third points, the association we found between the total adiponectin concentration and radiographic progression does not provide any direct indications about any functional roles of this adipokine in RA. Despite an anti-inflammatory role of adiponectin, adiponectin isoforms are proinflammatory on RA synovial cells, in accordance with our results [3]. Moreover, although adiponectin may be protective in collagen-induced arthritis, its proinflammatory effect is well known in other inflammation models [4].

Concerning the fourth point about potential discrepancies between a recently published cross-sectional study [5] and our own work, the comparison is challenging since we have not assessed the high molecular weight (HMW) isoform and have not investigated healthy control subjects. Moreover, Toussirot and colleagues did not study the structural progression. Finally, they investigated treated patients with established RA, while we focused on untreated patients with early RA. Furthermore, the use of an enzyme-linked immunosorbent assay for HMW assessment and a radioimmunoassay for total adiponectin assessment may explain the absence of correlation between both measurements. Recently, a high correlation between both isoforms using enzyme-linked immunosorbent assay for both measurements has been reported in RA [6].

Finally, we fully agree on discrepancies between published studies investigating the serum adiponectin level in RA as noted in the fifth point by Toussirot [1]. Although this can be due to the need to assess HMW rather than total adiponectin, the high correlation using an enzyme-linked immunosorbent assay does not support such a hypothesis. The demographic characteristics, the adjustment for confounding factors, and the sample size of the population may explain these divergences. Of note, our study involved the largest group of early RA adipokine measurements to date with multiple adjustments.

In conclusion, while our study has emphasized the usefulness of serum total adiponectin measurement as an accurate biomarker predicting radiographic progression, additional studies are necessary to establish whether the serum HMW adiponectin measurement may be more useful for such a purpose.

## Abbreviations

HMW: High molecular weight; RA: Rheumatoid arthritis.

## Competing interests

The authors declare that they have no competing interests.

## Author’s contributions

JS and FB wrote the letter. JC, J-PB and SF gave their advice about adiponectin isoforms and their assessment. All authors reviewed and approved the final manuscript.
